# PiGx: reproducible genomics analysis pipelines with GNU Guix

**DOI:** 10.1093/gigascience/giy123

**Published:** 2018-10-02

**Authors:** Ricardo Wurmus, Bora Uyar, Brendan Osberg, Vedran Franke, Alexander Gosdschan, Katarzyna Wreczycka, Jonathan Ronen, Altuna Akalin

**Affiliations:** Bioinformatics Platform, The Berlin Institute for Medical Systems Biology, Max-Delbrück Center for Molecular Medicine, Robert-Rössle-Strasse 10, 13125 Berlin, Germany

**Keywords:** pipelines in genomics, reproducible software, functional package management, RNA-seq, single cell RNA-seq, ChIP-seq, Bisulfite-seq, differential expression, differential binding, differential methylation

## Abstract

In bioinformatics, as well as other computationally intensive research fields, there is a need for workflows that can reliably produce consistent output, from known sources, independent of the software environment or configuration settings of the machine on which they are executed. Indeed, this is essential for controlled comparison between different observations and for the wider dissemination of workflows. However, providing this type of reproducibility and traceability is often complicated by the need to accommodate the myriad dependencies included in a larger body of software, each of which generally comes in various versions. Moreover, in many fields (bioinformatics being a prime example), these versions are subject to continual change due to rapidly evolving technologies, further complicating problems related to reproducibility. Here, we propose a principled approach for building analysis pipelines and managing their dependencies with GNU Guix. As a case study to demonstrate the utility of our approach, we present a set of highly reproducible pipelines called PiGx for the analysis of RNA sequencing, chromatin immunoprecipitation sequencing, bisulfite-treated DNA sequencing, and single-cell resolution RNA sequencing. All pipelines process raw experimental data and generate reports containing publication-ready plots and figures, with interactive report elements and standard observables. Users may install these highly reproducible packages and apply them to their own datasets without any special computational expertise beyond the use of the command line. We hope such a toolkit will provide immediate benefit to laboratory workers wishing to process their own datasets or bioinformaticians seeking to automate all, or parts of, their analyses. In the long term, we hope our approach to reproducibility will serve as a blueprint for reproducible workflows in other areas. Our pipelines, along with their corresponding documentation and sample reports, are available at http://bioinformatics.mdc-berlin.de/pigx

## Introduction

Reproducibility of scientific workflows is a ubiquitous problem in science and is particularly problematic in areas that depend heavily on computation and data analysis (see [1]). For such work, it is essential the installed software is identical to versions used in publication and be directly traceable to a well-defined set of source packages in order to facilitate the reproduction of published data and the controlled manipulation of these software systems. Unfortunately, this goal is often unattainable for a variety of related reasons. Research-oriented software may be hard to build and install due to unsatisfiable dependency constraints and nontrivial software may yield different results when built or used with different versions or variants of declared dependencies. On workstations and shared high-performance computing (HPC) systems alike, it may be undesirable or even impossible to comply with version and variant requirements due to software deployment limitations. Moreover, it is unrealistic to expect users to manually recreate environments that match the system and binary dependencies on which the software was developed. In the field of bioinformatics, the above problem is exacerbated by the fact that data production technology moves extremely fast; existing software and data analysis workflows require frequent updates. Thus, it is paramount that multiple versions and variants of the same software be automatically built in order to ensure reproducibility of projects that are either in-progress or are already published. Moreover, bioinformatics workflows are increasingly being applied to potentially sensitive medical data from research participants. For the sake of data security, it is important that researchers know exactly what sources are being used in an application in order to minimize the risk of code that might (either maliciously or inadvertently) compromise confidentiality [2]. Thus, bioinformatics represents a field where there is a need for both reproducibility and *r*eferential transparency (i.e., traceability to original software sources).

An important related issue is the reproducibility of workflows and pipelines across different machines. In addition to bioinformatics, many scientific fields require researchers to prototype their code on local workstations with a custom software stack and then later run it on shared HPC clusters for large datasets. The researcher must then be able to recreate their local environment on the cluster to ensure identical behavior. All of these concerns add to the burden on scientists, and valuable time that could be spent on research is wasted accommodating the limitations of system administration practices to ensure reproducibility. Even worse, reproducibility failures can be overlooked amid this complication, and publications could be accompanied with irreproducible analysis workflows or software. For these reasons, the scientific community, in general, and fast evolving fields like bioinformatics, in particular, need reliable and reproducible software package management systems.

In recent years, several tools have gained popularity among software developers and system administrators for wrapping Linux kernel features to accomplish process isolation, bind mounts, and user namespaces or to deploy services in isolated environments (also called “containers”). Examples of such tools include Docker, Singularity, and lxc. These tools are sometimes also proposed as solutions to the reproducibility problem [1, 3], because they provide a way to ship an application alongside all of its runtime dependencies. This approach necessitates the use of file system images that are modified using imperative statements, e.g., to run a package manager inside a namespace, with the goal of embedding all dependencies in an opaque binary image.

Such images, however, offer no indication as to the sources from which their contents originate. Although contributors following best practices will generally declare their dependencies, with many contributors, and inevitable human error, it can often become difficult to confidently ascertain the full contents of an opaque binary bundle. Software deployment inside of the container is still subject to the well-known limitations of traditional package managers, such as intractable stateful behavior, time-dependent installation results, and the inability to install and control more than a handful of application, or library, variants of packages on the same system, to name a few. Some of these limitations can partially be worked around by following strict policies such as operating version-controlled mirrors of all upstream package repositories. However, these policies are not enforced by container systems such as Docker. Rather, they only shift the problem of reproducibility from the package level to the level of binary disk images, a rather less useful level of abstraction.

Functional package management [4], on the other hand, embeds the complete dependency graph and configuration space into the construction of the package itself. This approach allows for referential transparency in addition to bit-for-bit build reproducibility. Other package and environment managers (such as Conda, EasyBuild, or Spack) leave out this information to varying degrees and rely on tacit assumptions about the deployment and build environments.

For the above reasons, we propose functional package management as implemented in GNU Guix [5]) as a way to implement workflow systems. To demonstrate the feasibility of this approach, we created a set of analysis tools (or “pipelines”) for common genomics analysis datasets: RNA sequencing (RNA-seq), chromatin immunoprecipitation sequencing (ChIP-seq), bisulfite-treated DNA sequencing (BS-seq), and single-cell resolution RNA sequencing (scRNA-seq). Each pipeline has a complex and large graph of dependencies, and each graph is comprehensively declared as a GNU Guix package definition. The graph is then built reproducibly by relying on Guix package manager features. Note that these pipelines also represent production-level pipeline tools, rather than simply model examples; they come with a full set of features including alignment, quality checking control, quantification, assay specific analysis, and HTML reports. This set of pipelines is referred to, collectively, with the acronym PiGx (for **P**ipelines **i**n **G**enomics^1^), pronounced “pigs.”

## Results

### Pipeline design and implementation philosophy

PiGx was designed with special focus on several key features, namely, that they be easy to use, easy to install, easy to distribute, reproducible, and referentially transparent, many of which are interrelated constraints. Care was taken to ensure that all of the pipelines have a similar interface, so that familiarity with one pipeline would make for a gentler learning curve in using the others. For the end user, each pipeline has the same input types: a sample sheet and a settings file. The sample sheet contains information about samples (such as names, labels, and covariates). The settings file contains extra arguments related to the execution of the pipelines. The users can generally run pipelines as follows:

pigx [pipeline_name] [sample_sheet] -s [settings_file]

where [pipeline_name] can refer to any of the four pipelines: “rnaseq,” “chipseq,” “bsseq,” or “scrnaseq.” The resulting output provided to the users includes high-quality reports and figures containing a standard set of results from basic analyses and data quality checks. Where appropriate, reports also contain certain interactive elements.

In implementing this toolset, one of our first design choices was to use a conventional build system, the GNU Autotools suite, to configure and build the pipelines as if they were first-class software packages in their own right rather than a mere collection of tools and “glue code.” Instead of assuming that a user will provide a suitable environment at runtime, the use of a build system allows us to capture the software environment at configuration time. This is achieved by explicitly checking for the presence of required tools in the build environment and recording their exact location in the pipeline's configuration file. At runtime, the pipeline refers only to tools through the configuration file and does not assume the availability of dependent software in the global environment. Moreover, using a well-established build system makes it easy to package the pipelines for any package manager. We chose GNU Autotools over other build systems for two reasons: it does not require users to have a copy of the build system software as it compiles to shell code (which is highly portable) and it has been established long enough to implement a conventional and flexible build interface with well-known behavior even in somewhat unusual circumstances, such as the installation of files into unique prefixes as is done when building with GNU Guix.

Capturing the build-time environment alone is not enough to ensure reproducibility, nor is the use of a build system sufficient to make installation easy. Thus, our second design choice was to package the pipelines for the GNU Guix package manager. Like other user-level package managers such as Conda or EasyBuild, GNU Guix allows users to install, upgrade, and remove software without having to know the details of dependencies or the build procedure. Unlike traditional package managers, however, GNU Guix takes a declarative approach to software environments called "functional package management." This approach takes into account the complete graph of dependencies and build-time configurations and maximizes build reproducibility by building binaries in isolated environments. Packages are installed into directories with unique prefixes that are computed from the dependency graph, allowing for the simultaneous installation of different versions or variants of applications and libraries. With functional package management, a given software build will generally yield bit-identical files when the build is performed on different machines or on the same machine at different points in time, independent of the current state of the system (caveats to this generalization are discussed below).

We consider software reproducibility an important asset in controlled experimentation. Reproducing a software environment bit for bit is not a goal in itself but it provides us with a foundation upon which we can perform precise changes to the environment and assess the impact of these changes. Without bit-for-bit reproducibility, we cannot be certain of the nature and impact of differences in the software environment. While virtual machines or binary application bundles such as Docker images would be sufficient to freeze the state of our software environment, relying on these tools would forgo the ability to recreate that same environment from scratch, and it would not be possible to analyze the environment at the level of software packages. The approach of functional package management as implemented in GNU Guix preserves the relationships between software packages and ensures that differences to the environment can be accounted for.

A further design choice remained regarding the workflow management system, which would execute a series of tasks mostly in the form of scripts from different programming languages. For this purpose, we used SnakeMake [6], which provides target-driven execution infrastructure similar to GNU Make but with Python syntax, along with useful features such as parallel execution on HPC scheduling systems. We would like to emphasize, however, that this choice of workflow management system was made purely to facilitate ease of development and acceptance within the bioinformatics community, where the Python programming language is well established. The different pipeline stages are implemented with a workflow management system stitching together various bioinformatics tools; they are made configurable with the GNU Autotools and packaged with GNU Guix. This means they will be almost fully (see below) build-reproducible and can be installed via the one-liner:

guix package -install pigx.

### RNA-seq pipeline

#### General description of PiGx-RNA-seq pipeline

PiGx RNA-seq provides an end-to-end preprocessing and analysis pipeline for RNA-seq experiments. The pipeline takes a set of raw fastq read files and the experimental design as described by the user and produces differential expression reports with figures and tables of differentially expressed genes, as well as Gene Ontology (GO) term analysis thereof. Furthermore, it provides quality control reports about the experiment. To use the pipeline, the user must provide two files: the sample sheet describing the samples and corresponding fastq files and a settings file with configuration parameters related to the pipeline's execution. The settings file lists, among other things, the location of a reference genome for alignment, a GTF file with genome annotations, and a transcriptome reference, as well as a list of desired differential expression analyses to be performed, specifying which samples to use as cases and controls (see package documentation here [7] for more details).

**Figure 1:**
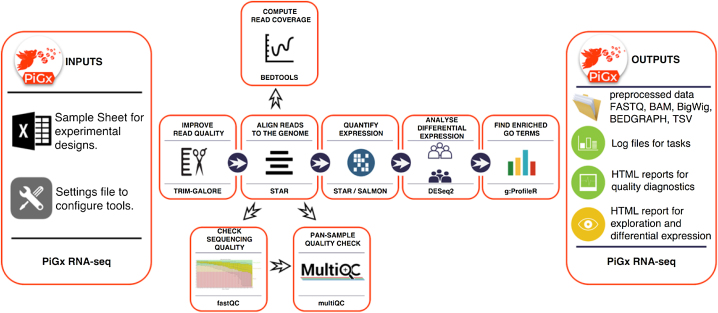
Workflow diagram of the PiGx RNA-seq pipeline.

The pipeline can then be run with the command pigx rnaseq [sample_sheet] -s [settings_file] to generate the output through several intermediate steps (see Fig. 1).

PiGx RNA-seq uses the reference genome and transcriptome provided by the user to produce indices using *STAR* [8] and *Salmon* [9], respectively. It then uses *Trim Galore!* [10] to trim low-quality reads and remove adapter sequences before aligning the reads to the reference using *STAR*. At this point, PiGx RNA-seq uses *fastqc* [11] and *MultiQC* [12] to generate comprehensive quality control reports of the sequencing, trimming, and alignment steps. PiGx RNA-seq also uses *BEDTools* [13] to compute the depth of coverage in the experiment and outputs convenient bedgraph files. Gene expression quantification is obtained from *STAR* and transcript-level quantification using *Salmon*. The gene expression count matrix is then used to run differential expression analyses, as specified by the user, using *DESeq2* [14] for statistical analysis and *g: ProfileR* [15] for GO term analysis. Each differential expression analysis produces a self-contained HTML report.

The differential expression reports produced are comprehensive, including sortable tables for differentially expressed genes for a detailed view, principal component analysis plots for a bird's-eye view of the experiment, as well as MA and volcano plots. In addition, the reports include a section with GO term enrichment analysis.

#### RNA-seq use case

The study by Hon et al. [16] is motivated by several observations: DNA methyl-transferases (DNMTs) are the major mediators of cytosine methylation (producing 5-methyl-cytosine [5mC]); 5-hydroxy-methyl-cytosine (5hmC) is a product of oxidation of 5mCs; and the TET family of proteins mediate 5mC oxidation. It has been established that DNA demethylation consists of the sequence of chemical reactions that convert 5mC into 5hmC, which is subsequently converted into 5-formyl-cytosine (5fC) and 5-carboxyl-cytosine (5caC). Active enhancers are depleted for 5mC but are enriched for 5hmC marks [17], suggesting that an interplay between DNMTs and TET proteins could determine the activity level of enhancers. Mutating DNMTs or TET proteins in mouse embryonic stem cells (mESCs) perturbs global DNA methylation status; however, cells do not lose the ability to regenerate. Moreover, mutating TET proteins and perturbing the oxidation levels have previously been shown to skew the differentiation of mESCs. Based on these facts, the authors address the following question: can the skewed differentiation in mESCs be explained by deregulated balance of 5mC/5hmC levels at active enhancers following the loss of activity of TET proteins?

The authors of the above study use TAB-Seq, BS-Seq, ChIP-seq, and RNA-seq methods to profile genome-wide methylation, demethylation, histone modifications, and gene expression levels to address these questions. They find that *Tet2* has the biggest role in enhancer demethylation in mESCs. Deletion of *Tet2* leads to enhancer hypermethylation, which in turn reduces enhancer activity. The reduced enhancer activity leads to a disruption in the activation of more than 300 genes in the early stages of differentiation; however, the activity levels of these genes are restored to wild-type levels at the later stages of differentiation. Reduced enhancer activity followed by delayed gene activation explains the skew observed in mESC differentiation.

The authors of the above study profile the transcriptomes of mESCs as they differentiate into neural progenitor cells within a 6-day period. They quantified gene expression levels of wild-type, *Tet1* -/-, and *Tet2* -/- cells on day zero, day 3, and day 6 and sequenced two biological replicates per sample. Thus, they obtained 18 samples in total (3 genotypes x 2 replicates x 3 days). In Fig. 5 of the original manuscript, the authors summarize the results of the RNA-seq analysis. Here, we use the PiGx-RNA-seq pipeline to preprocess the raw fastq files downloaded from the GEO archive (GEO accession: GSE48519), map the reads to the *Mus musculus* genome (GRCM38 [mm10] build), and finally quantify the expression levels of genes using both Salmon [9] and STAR [8]. We then use DESeq2 [14] to perform multiple differential expression analyses as described in the original publication. Based on the processed and normalized count tables and differential expression analysis results produced by the PiGx pipeline, we have written a small custom script to reproduce the panels in Fig. 5 of Hon et al. In order to reproduce this figure, we needed to perform seven differential expression analyses as described in Table 1. HTML reports for each differential expression analysis (based on read counts computing using STAR) can be found in [18].

**Table 1:** Differential expression analyses performed by PiGx-RNA-seq

Analysis	Case sample	Control sample	Description
tet2_diff_day3	day3_tet2_KO	day0_tet2_KO	*Tet2* -/- cells on day 3 are compared to *Tet2* -/- cells on day 0.
tet2_diff_day6	day6_tet2_KO	day0_tet2_KO	*Tet2* -/- cells on day 6 are compared to *Tet2* -/- cells on day 0.
WT_diff_day3	day3_WT	day0_WT	Wild-type cells on day 3 are compared to wild-type cells on day 0.
WT_diff_day6	day6_WT	day0_WT	Wild-type cells on day 6 are compared to wild-type cells on day 0.
tet2_vs_WT_day0	day0_tet2_KO	day0_WT	*Tet2* -/- cells on day 0 are compared to wild-type cells on day 0.
tet2_vs_WT_day3	day3_tet2_KO	day3_WT	*Tet2* -/- cells on day 3 are compared to wild-type cells on day 3.
tet2_vs_WT_day6	day6_tet2_KO	day6_WT	*Tet2* -/- cells on day 6 are compared to wild-type cells on day 6.

**Figure 2:**
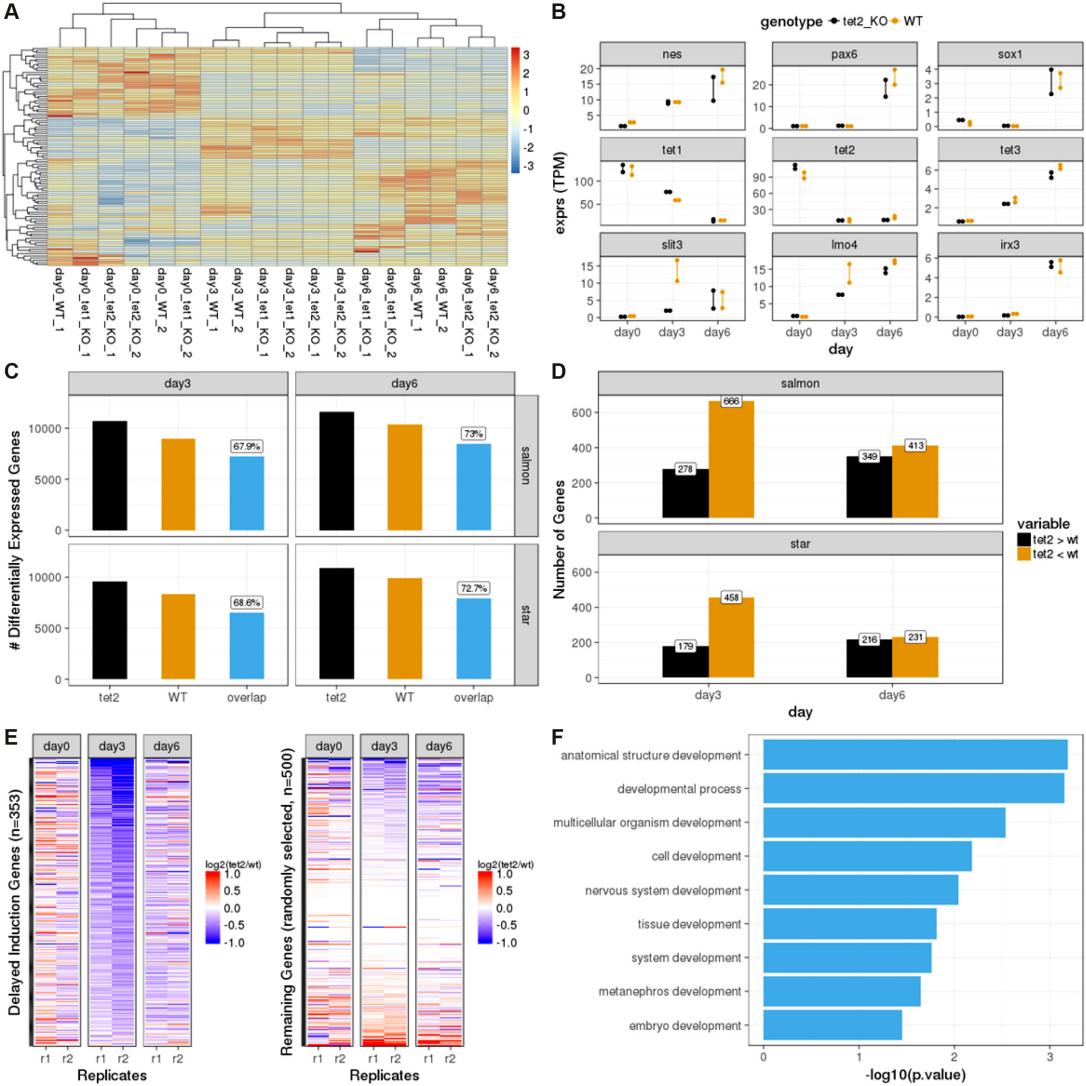
Reproduction of . 5 from [16] using datasets processed by the PiGx-RNA-seq pipeline. **(A)** Hierarchically clustered heat map of the top 100 most variable genes across all samples (transcripts per million [TPM] aggregated on the gene level, produced with Salmon). Each row represents a gene and each column represents a sequenced sample (see Table 1 for descriptions of the samples). The expression values are scaled by "row.". **(B)** Changes in the expression levels of a selected list of genes throughout the differentiation period on day 0, day 3, and day 6. The *y*-axis shows the normalized expression levels (TPM at the gene level). The expression patterns of samples with *Tet2* -/- background are depicted in black and wild-type background in orange. **(C)** Abundance of differentially expressed genes (adjusted *P* value < 0.1) (on *y*-axis) when comparing samples on day 3 or day 6 with the samples on day 0 with corresponding genotypes (*Tet2* -/- or wild type). The bar labeled “overlap” represents the number of differentially expressed genes in both genotypes. The percentage is calculated by dividing the value of “overlap” with the value of *Tet2*. The results are reproduced by both Salmon-based gene-level read counts (top row) and STAR-based gene-level read counts (bottom row). **(D)** Genes that are upregulated (induced) in wild-type samples on day 3 (or day 6), compared to wild-type samples on day 0, are intersected with genes that are differentially expressed between wild-type samples and *Tet2* -/- samples at the same stage of differentiation and classified as *“Tet2* > wt*”* (the gene is upregulated in the *Tet2* -/- sample more so than in the wild-type sample) or *“Tet2* < wt*”* (the gene is upregulated in *Tet2* -/- sample less than in the wild-type sample). The plot is reproduced using both Salmon-based gene counts and STAR-based gene counts. **(E)** Heat maps for delayed induction genes (on the left) and 500 genes randomly selected from the remainder (on the right). The colors of the heat map represent the log_2_ scale ratio of normalized expression value (gene-level TPM counts obtained using Salmon) of each delayed induction gene between *Tet2* -/- sample and the wild-type sample of the corresponding replicates (r1: replicate-1, r2: replicate-2) on the corresponding stages of differentiation (day 0, day 3, and day 6). The rows of the heat map are ordered in increasing order based on the average values of the two replicates on day 3. The color scales range between –1 and 1 before reaching saturation. **(F)** Top GO terms for biological processes (on the *y*-axis) enriched among the delayed induction genes. The GO terms are detected using g: ProfileR tool[15]. The resulting terms are filtered for *P* value <0.05 and further filtered for the keyword “development.” On the *x*-axis, the *P* values are depicted at log_10_ scale.

Having performed the above analysis, we first took a global look at how all sequenced samples cluster. Using a table of transcripts per million reads (TPM) counts generated by Salmon at the gene level, we selected the top 100 most variable genes and plotted a heat map of all the samples using the pheatmap package [19]. We observed that the samples mainly cluster by the differentiation stage rather than genotype, which confirms the authors' findings (Fig. 2A). Next, again using the same TPM counts table, we plotted the expression levels of a select list of genes (*Nes6, Pax6, Sox1, Tet1, Tet2, Tet3, Slit3, Lmo4, Irx3*) on day 0, day 3, and day 6 (Fig. 2B). The changes in the expression levels of these genes perfectly match the patterns described by Hon et al. At this point, the authors recognize that some neural marker genes, such as *slit3* and *lmo4*, show discordant expression patterns between WT and *Tet2* -/- samples, particularly on day 3, which are restored back to WT levels on day 6. The authors then determine whether such a delayed induction mechanism can be observed globally. It was shown that the percentage of genes that are differentially expressed in both *Tet2* -/- and WT cells (compared to the undifferentiated samples of the corresponding genotypes on day 0) is significantly higher on day 6 than on day 3. We also observe a similar pattern; however, the difference we observe is somewhat reduced. Our findings are reproduced based on gene counts quantified by both STAR and Salmon (Fig. 2C).

**Figure 3:**
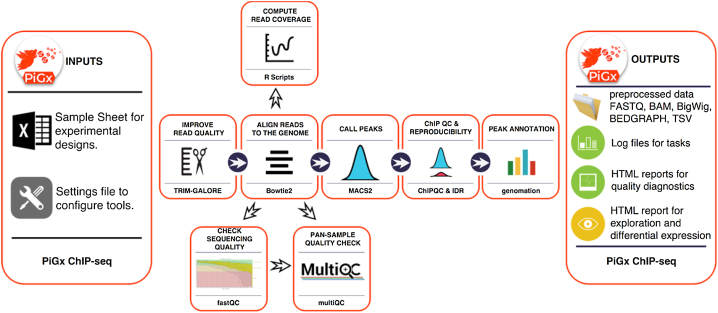
Workflow diagram for ChIP-seq pipeline.

In Fig. 5F of the original publication, the authors take a closer look at the list of discordantly induced genes on day 3 in *Tet2* -/- samples. There it is shown that the majority of the genes that get induced in WT samples by day 3 do not get induced in the *Tet2* -/- samples as highly as they do in the WT samples. On the other hand, these numbers are comparable on day 6. We also observe the same difference and reproduce the findings using both Salmon and STAR-based gene counts (Fig. 2D). This suggests that there must be a list of genes that get activated in WT but lag behind in *Tet2* -/- samples at the early stage of differentiation; however, they catch up later with the WT levels. The authors call these genes “delayed induction genes” and find 333 genes that fit such a description. In Fig. 5G, the authors show the relative expression of these genes in *Tet2* -/- samples compared to WT samples throughout differentiation and compare it to the remaining list of genes in the genome. We have successfully reproduced the same patterns based on 357 delayed induction genes detected by Salmon-based gene counts (282 genes detected by STAR-based gene counts) (Fig. 2E). In Fig. 5H, the authors show the most significant GO terms enriched for the delayed induction genes. Although we do not observe the same set of terms as reported by the authors, we found seven development-related GO terms, including “tissue development” and “nervous system development,” as enriched terms (Fig. 2F).

### ChIP-seq pipeline

#### General description of PiGx-ChIP-seq pipeline

PiGx ChIP-seq is an end-to-end processing and analysis pipeline for ChIP-seq experiments (See Fig. 3). From the input fastq files, the pipeline produces sequencing quality control, ChIP quality control, peak calling, and IDR (Irreproducoble discovery rate) [20] estimation. PiGx ChIP-seq also prepares the data for visualization in a genome browser. The pipeline execution is highly customizable; the user can specify which parts of the pipeline to execute and which parameter settings to use. As in the other pipelines, to use PiGx ChIP-seq, the user must provide two files: a sample sheet containing the names of the fastq files with a descriptive label and a settings file. The settings file contains the locations of the reference genome and the GTF file with genome annotations, as well as a list of configurations for each executable step. Upon completion, the user is provided with quality reports and all of the preprocessed data, which substantially facilitate downstream analysis and visualization.

The pipeline can then be run with the command:

pigx chipseq [sample_sheet] -s [settings_file]

PiGx ChIP-seq pipeline aligns the reads to the genome using *Bowtie2* [21], does peak calling using *MACS2* [22], calculates the irreproducibility rate, and outputs a series of quality statistics, such as GC content, strand cross-correlation, distribution of reads and peaks over annotated genomic features, and clustering of samples based on their similarity [23]. The pipeline also produces UCSC Track hubs to facilitate exploration of the dataset. The purpose of the pipeline is to improve the routine processing steps for ChIP-seq experiments and enable the user to focus on data quality control and biologically relevant data exploration. The pipeline heavily depends on Bioconductor [24] packages such as GenomicRanges [25] and Genomation [26] for annotating peaks and summarizing ChIP-seq scores over regions of interest.

#### ChIP-seq use case

**Figure 4:**
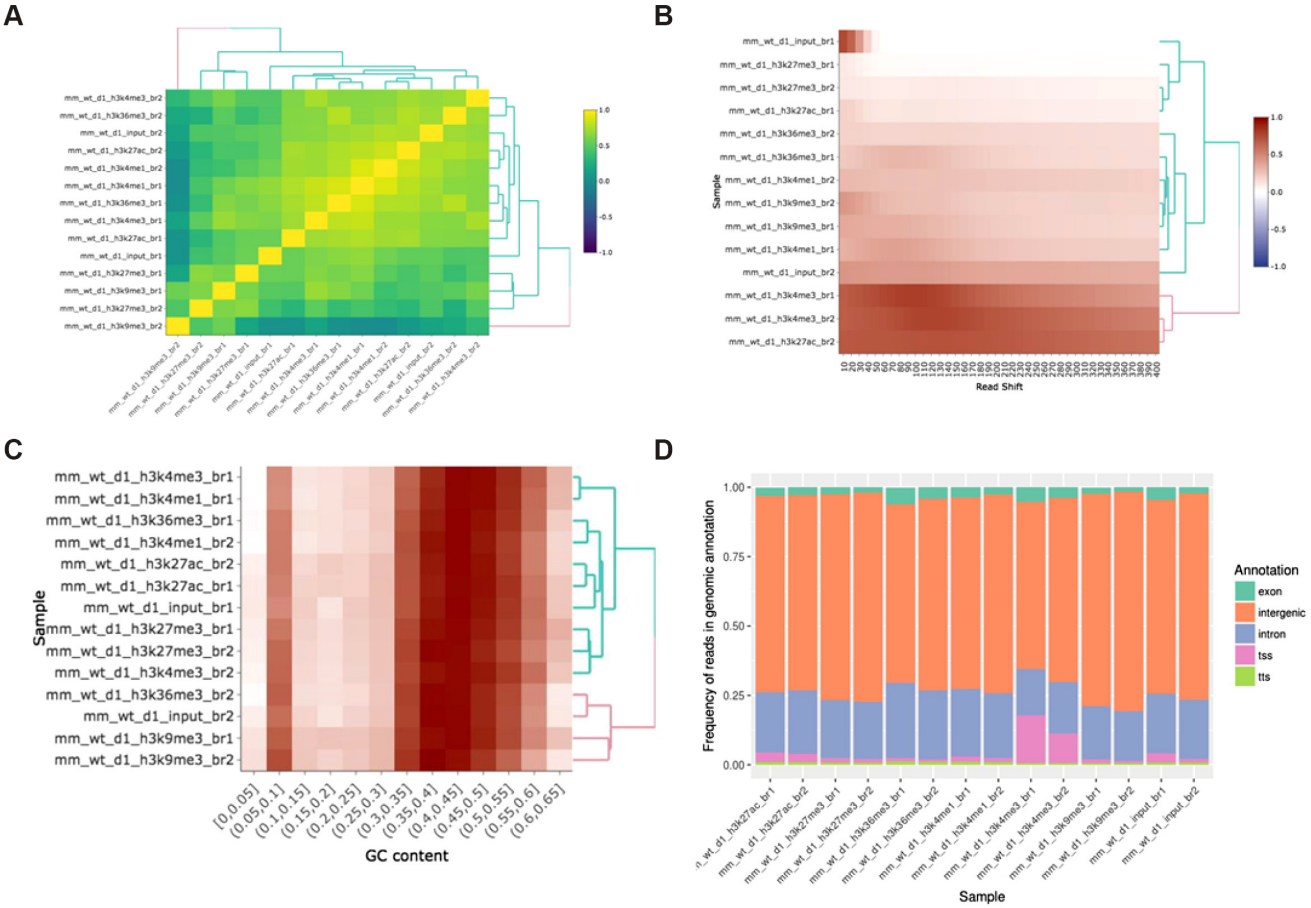
Example ChIP-seq quality control output. **(A)** Clustering of samples based on correlation of normalized ChIP reads in 1 kb bins. **(B)** Cross-correlation between coverage profiles on Watson and Crick strands, shifted by the amount specified on the *x*-axis. **(C)** Relationship between read count and GC content in 1 kb bins. **(D)** Distribution of reads in functional genomic features.

For consistency, we applied the ChIP-seq pipeline to data from the same study as in the section “RNA-seq Use Case” above [16]; for the biological underpinnings of this experiment, please see the description provided there. Figure 4 shows part of the ChIP-seq quality control output performed on untreated, wild-type ChIP samples of various activating and repressing histone marks and the corresponding input samples. One standard procedure is to validate the consistency of results with known biological priors in order to quickly find samples with outlying properties and to discover batch effects. For example, Fig. 4A shows the expected clustering of repressive (H3k27me3, H3k9me3) and activating (H3k4me3, H3k4me1, H3k27ac, and H4k36ac) histone marks. Upon closer inspection, however, it becomes clear that the activating histone marks cluster by their corresponding *batches*, and not by their biological functionality.

**Figure 5:**
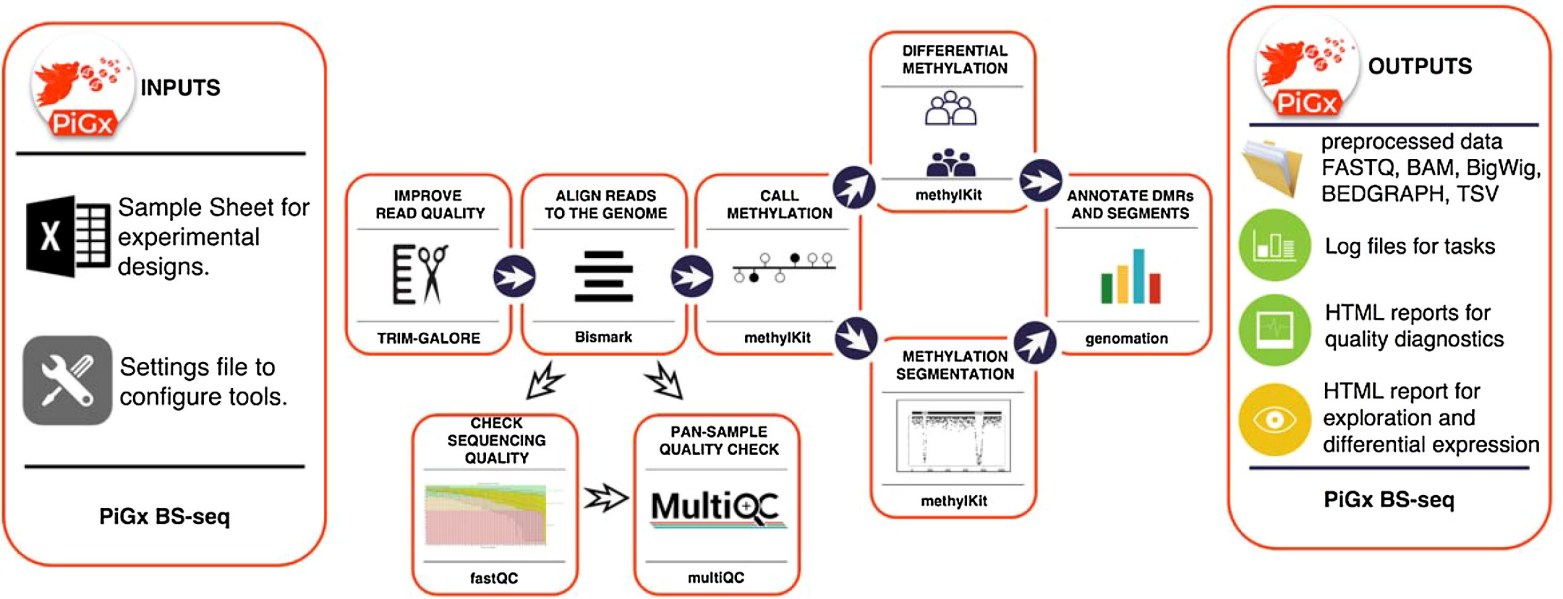
Workflow diagram for PiGx BS-seq pipeline.

Figure 4B shows the cross-correlation between the signal on the plus and minus genomic strands, shifted by a defined range (usually within a range of 1 to 400 nucleotides). The maximum intensity in each row indicates the average DNA fragment size in each corresponding ChIP experiment. Large discrepancies in the cross-correlation profile, between experiments, can indicate problems with fragmentation, fixation, or chromatin immunoprecipitation. The figure shows that most of the samples have an average fragment size of between 100 and 150 bp. One of the H3k27me3 replicates, however, shows an aberrant fragment size profile (second sample in the plot). Upon visual inspection, the sample had an extremely low signal-to-noise ratio and the peak calling resulted in zero enriched regions. Such samples should either be repeated or omitted from the downstream analysis.

Figure 4C represents the relationship between the GC content of 1 kb genomic bins and the ChIP signal. This plot is used as a diagnostics tool for enrichment of fragments with extreme nucleotide content (enrichment of fragments with GC content strongly deviating from the genomic mean), which can indicate problems with polymerase chain reaction-based fragment amplification and chromatin immunoprecipitation.

Figure 4D represents the distribution of reads over functional genomic features. It is used to determine whether the experimental results conform to known expectations, based on previous experiments, i.e., H3k4me3 should show strong enrichment over transcription start sites, while the H3k36me3 should show an enrichment over exonic and intronic regions. Deviating results can indicate a weak precipitation of the targeted protein or antibody cross-reactivity with unexpected epitopes. Figure 4 represents just a subset of quality control metrics implemented as a standard output from the PiGx ChIP-seq pipeline. The full set can be found in [18].

### BS-seq pipeline

#### General description of the PiGx BS-seq pipeline

PiGx BS-seq is a bisulfite sequencing processing pipeline used to detect genome-wide methylation patterns and to perform differential methylation calling for case-control settings (See Fig. 5). It produces individual reports for each sample provided by the user, in addition to differential-methylation reports for arbitrarily many pairs of treatment conditions provided by the user. PiGx BS-seq uses *Trim Galore!* [10] to trim reads for adapter sequences and quality and *fastqc* [11] for quality control (both before and after trimming). If necessary, PiGx BS-seq produces *GA*- and *CT*-converted versions of the reference genome for alignment, using bismark_genome_preparation [27]. Reads are then mapped to the reference using Bowtie2 [21] before being sorted by location in the genome and filtered for uniqueness using samtools [27, 28]. The corresponding reports and .bam files for each of these steps are saved to their respective directories.

As in the other pipelines, to use PiGx BS-seq, the user must provide two input files: a sample sheet containing the paths to the fastq files with a descriptive label and a settings file. The pipeline is robust to paired-end or single-end input data, and processing of each case is initiated automatically based on whether the user supplies only a single input file or a pair of files for a given sample. The settings file contains the locations of the reference genome, among other directories, as well as a list of configuration steps for each executable step. The pipeline can then be run with the command:

pigx bsseq [sample_sheet] -s [settings_file]

Post-mapping analysis steps performed automatically by PiGx BS-seq include tabulation of the fractional methylation of CpG sites, the segmentation of genomic methylation patterns across the genome, and the selection of differentially methylated sites between pairs of treatments provided in the settings file above. Furthermore, the final reports include genomic annotation of differentially methylated regions and methylome segments. A single execution of the pipeline can perform differential methylation analysis between a sample and arbitrarily many references; each comparison will have its own dedicated report, in addition to the final report for the sample itself. For traceability, direct links to input files and various execution tools are saved directly within the output folder. Finally, a copy of the full methylome for each sample is also saved in BigWig (.bw) format, compatible with visualization in an online genome browser.

#### BS-seq use case

We applied the BS-seq pipeline to data from embryonic stem cells in mice, comparing wild-type and *Tet2* deletion experiments (accessions SRX317877 and SRX317883, respectively). These datasets derive from the same study as was used for controlled comparison in the section “RNA-seq Use Case” above [16]. For a biological description of this experiment, please refer to that section. HTML reports for each of the performed analyses can be found in [18].

**Figure 6:**
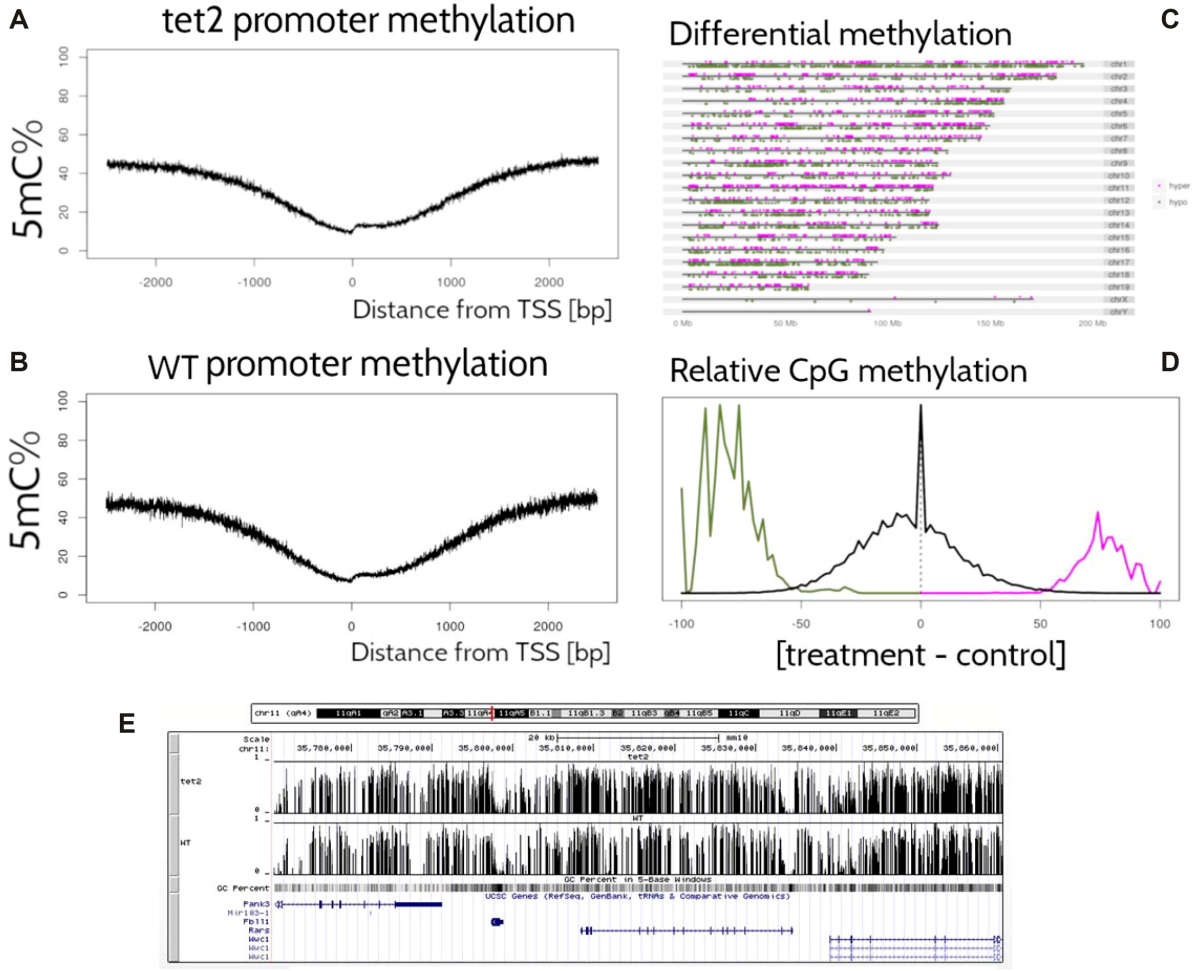
Output from the PiGx BS-seq pipeline. **(A, B)** Average CpG methylation throughout the promoter regions of the mm10 genome for *Tet2* -/- and WT, respectively, as a function of distance from TSS (in direction of transcription). **(C)** Whole-genome map of differentially methylated CpGs, with color code to indicate hyper- and hypomethylation of the treatment (*Tet2* -/-) relative to the control (wild-type). **(D)** Histogram of the difference in average CpG methylation between *Tet2* -/- and wild-type. For differentially methylated cytosines, colors are consistent with (C), while CpGs with statistically insignificant difference in methylation is provided in black. Normalization of these two curves is performed independently (since the latter are generally far more numerous than the former), and the graph conveys only relative proportions (thus, as the absolute *y*-axis is of arbitrary scale, units are omitted). **(E)** Screenshot of the genome browser using bigwig data from PiGx. Here, the data can be examined in much finer detail than in (C).

Figure 6 shows a standard set of data analysis metrics generated automatically by the pipeline. For example, methylation levels near the promoter region of a list of annotated genes for each sample are shown in Fig. 6A and 6B. For generality, Fig. 6 averages over all known genes; however, the user may freely probe for more specific results by supplying any arbitrary set of genes under investigation (in the absence of such an annotation file, this figure is simply omitted from the final report). A coarse map of the genome is provided in (Fig. 6C, which, for some datasets, may serve to highlight differential methylation localized to particular regions or chromosomes. In this particular use case, it is more useful as a null control showing that these regions are uniformly distributed throughout the genome. In addition, a histogram for differential methylation status of CpGs throughout the genome is provided in Fig. 6D using the same color code as in Fig. 6C. The methylation differences of hypermethylated, hypomethylated, and nondifferentially methylated CpGs are shown as histograms with the color code as in Fig. 6C. The latter is shown as a distribution of methylation differences deemed to be not statistically significant (in black). Since these are generally far more numerous than the former, the two curves are normalized independently. Note also that since these curves represent relative distributions, the vertical axis is of arbitrary units and tick marks are omitted. Finally, a screenshot of data visualization from the genome browser [29, 30] is provided in Fig. 6E. Here, regions of interest can be inspected manually at arbitrary precision.

### scRNA-seq pipeline

#### General description of the PiGx scRNA-seq pipeline

**Figure 7:**
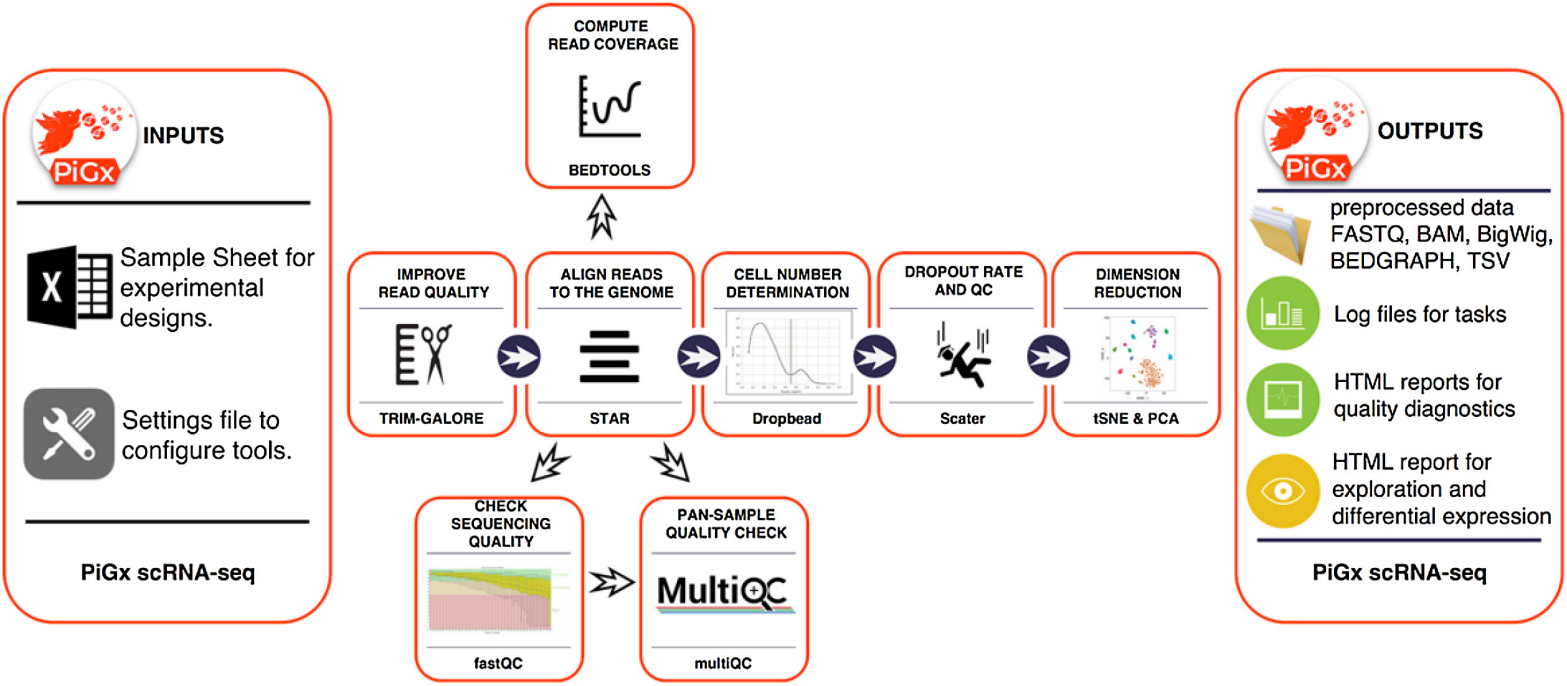
Workflow diagram for PiGx scRNA-seq pipeline.

scRNA-seq is an extremely powerful technology that is becoming increasingly prevalent in biological studies. The rapid development of unique molecular identifier (UMI)-based methods, along with droplet-based cell separation [31, 32], has enabled even simple experiments to quantify expression in several tens of thousand of cells. PiGx scRNA-seq is a pipeline for preprocessing of UMI-based single-cell experiments (See Fig. 7). The purpose of the pipeline is to enable seamless integration and quality control of multiple single-cell datasets. The pipeline works with minimal user input. As in the other pipelines, the user must provide a sample sheet with a basic experimental description and a settings file that defines, among other parameters, the location of the input data and reference sequence and annotation. The pipeline can then be run with the command:

pigx scrnaseq [sample_sheet] -s [settings_file]

The pipeline does preliminary read processing, maps the reads with the STAR [8] aligner, and assigns reads to gene models. It also separates cells from background bar codes [33] and constructs digital expression matrices for each sample (each saved in loom format); loom files from all samples are then merged into one large loom file using the loompy package [34]. The expression data are subsequently processed into a SingleCellExperiment (Aaron [35]) object. SingleCellExperiment is a Bioconductor class for storing expression values, along with the cell and gene data, and experimental meta data in a single container. It is constructed on top of hdf5 file-based arrays [36], which enables exploration even on systems with limited random access memory.

During the object construction, the pipeline performs expression normalization, dimensionality reduction, and identification of significantly variable genes. The pipeline then classifies cells by cell cycle phase and calculates the quality statistics. The SingleCellExperiment object contains all of the data needed for further exploration. The object connects the PiGx pipeline with the Bioconductor single-cell computing environment and enables integration with state-of-the-art statistical and machine learning methods (scran [37], zinbwave [38], netSmooth [39], iSEE [40], etc.).

The pipeline produces an HTML report containing quality controls, labeled by input covariates, that can be used for detecting batch effects.

#### scRNA-seq use case

**Figure 8:**
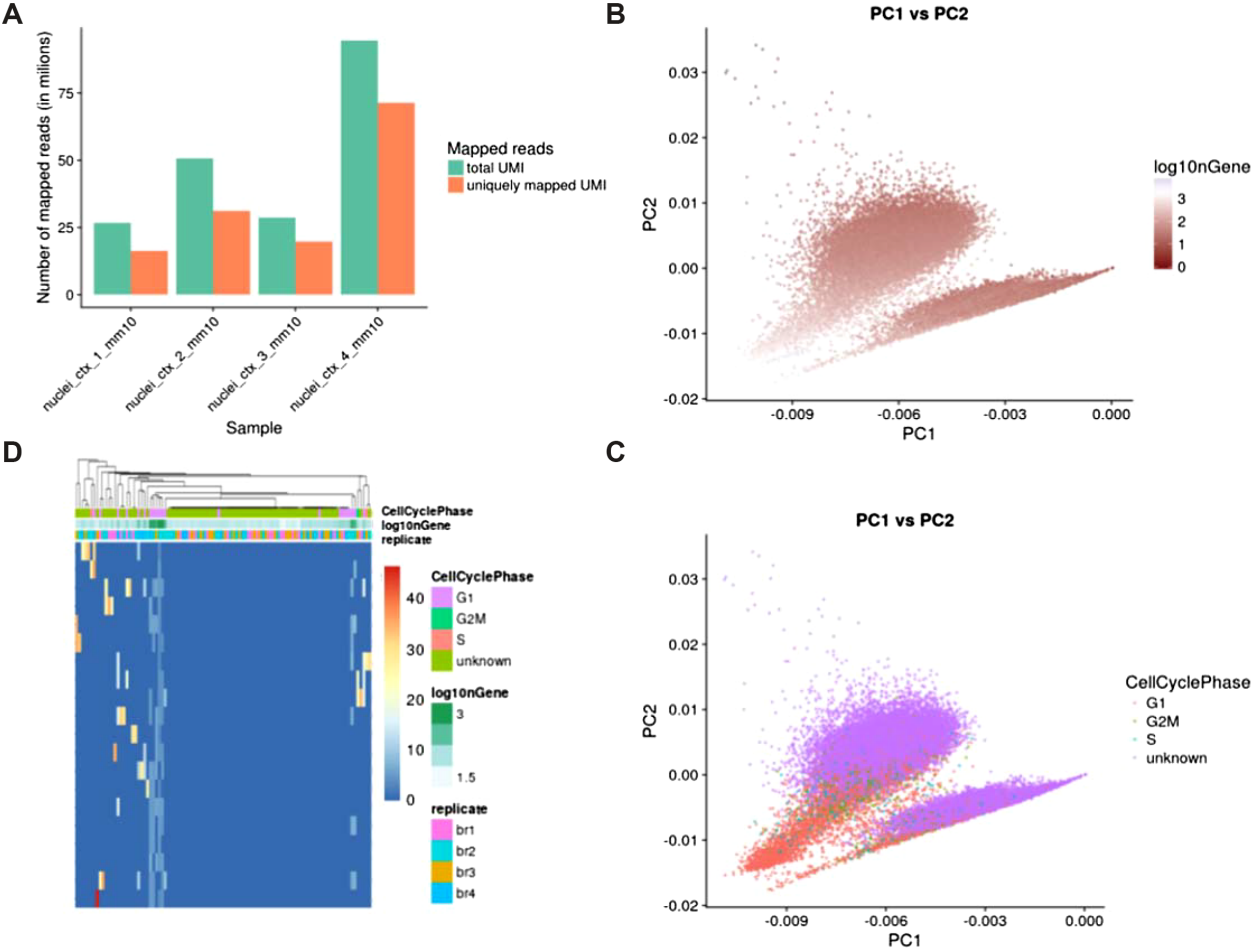
Sample output from the PiGx scRNA-seq pipeline. **(A)** Abundance of total uniquely mapping UMIs per sample. **(B)** Visualization of cells on the first and second principal component calculated from the normalized expression values. The gradient shows the total number of UMIs per cell. **(C)** Same data representation as in B but colored based on the cell cycle assignment. Cell cycle was assigned using the cyclone function from the scran Bioconductor package [37]. **(D)** Expression heat map of genes contributing most to the first principle component. Genes are ordered in rows, while cell are in columns. Color bars above the heat map show relevant experimental variables.

To showcase the capabilities of PiGx scRNA-seq, we ran the pipeline on isolated single nuclei from the mouse brain [41]. In this study, the authors developed a gradient-based method for nucleus separation and used it in combination with Drop-seq to profile the transcriptomes of more than 18,000 single nuclei. Figure 8 shows a part of the quality control output from the PiGx scRNA-seq pipeline. Figure 8A shows the per sample number of total and uniquely mapped reads. Figure 8B visualizes the cells on the first two principal components. The color gradient corresponds to the number of detected genes per cell. The figure shows that the total number of detected genes strongly correlates with the first two principal components. Figure 8C is analogous to Fig. 7B of the original publication, with the color scheme representing labeling each cell with its respective stage of the cell cycle. Thus, Fig. 8C shows that the first two principal components correlate with the stage of the cell cycle. The heat map in Fig. 8D shows scaled normalized expression values for genes that contribute the most to the first principle component. High read-count variability in a small number of genes drives the variation around the first principle component. The column-wise annotations show that the variation is driven mainly by cells in the G1 phase of the cell cycle from the second biological replicate. The HTML report for this analysis can be accessed at [18].

### Reproducibility metrics of the pipelines in different systems

We define the complete software environment needed for each of the pipelines using Guix package definitions. These package specifications not only outline the immediate dependencies of the pipelines, they also extend to the full software stack recursively. The dependency graph is rooted in a handful of bootstrap binaries. Apart from these binary roots, every application or library in the graph is built from source. Guix ensures that packages are built in an isolated environment in which nothing but the specified dependencies are available. This is a precondition for bit-reproducible builds, i.e., repeatable package builds that yield the very same binary output for the same set of inputs. Under ideal circumstances (see below), a Guix specification for the complete dependency graph and the set of all source code would be sufficient to exactly reproduce the very same binaries of the pipelines presented here.

Unfortunately, there are additional obstacles to bit reproducibility that cannot be avoided purely by the functional package management model. Examples for sources of irreproducibility in build artifacts include embedded time stamps, nondeterministic sorting of strings, and nondeterministic compiler output. While some of these obstacles can be removed by deliberate patching of compilers or applications, others are harder to diagnose and can thus lead to failure to reproduce the same arrangement of bits in independent builds, be that on the same machine at different points in time or on different systems. In the reports produced by our pipelines, we can eliminate differences due to time stamps by controlling them with the SOURCE_DATE_EPOCH environment variable. This option can be invoked in order to produce identical HTML reports, provided there are no tools that introduce nondeterminism (as is the case for the PiGx BS-seq pipeline).

To estimate the level of bit reproducibility in our pipelines, we checked out version v0.14.0–3597-g17967d1 of GNU Guix, repeatedly built the pipeline packages pigx-rnaseq, pigx-bsseq, pigx-chipseq, pigx-scrnaseq and their direct dependencies on three different systems (an office workstation, a virtual machine, and a build farm consisting of 20 heterogeneous build nodes), and recorded the hashes of the package trees that were produced. Whenever the hashes of any two builds differed, we looked at the exact differences with diffoscope [42]. Upon closer inspection, we identified a number of common issues in nondeterministic builds, such as time stamps embedded in compiled binaries and text files and randomized file names in files generated by test suites.

Python dependencies are of particular note here because they are generally not reproducible due to the fact that the byte compiler records the time stamp of the source file in the compiled binary. This means that all compiled Python files will differ when they are compiled at different points in time. (This problem will be addressed in the upcoming Python 3.7, which will implement PEP 552 for deterministic compilation.) To avoid this problem and increase the number of packages that could be made reproducible, we patched our variant of Python 3.6 such that it resets the embedded time stamp in compiled files to the Unix epoch. This allowed us to greatly increase the number of fully bit-reproducible packages. As can be seen in Table 2, only 8 of 355 packages (or about 2.2%) were not bit reproducible for as-yet unknown reasons.

**Table 2:** Number of dependent packages and their reproducibility status

Package	Not reproducible	Minor problems	Reproducible
pigx-bsseq	2	2	167
pigx-chipseq	7	9	236
pigx-rnaseq	7	9	211
pigx-scrnaseq	6	8	218
All pipelines	8	9	338

See Table 3 for more details about packages with minor problems.

**Figure 9:**
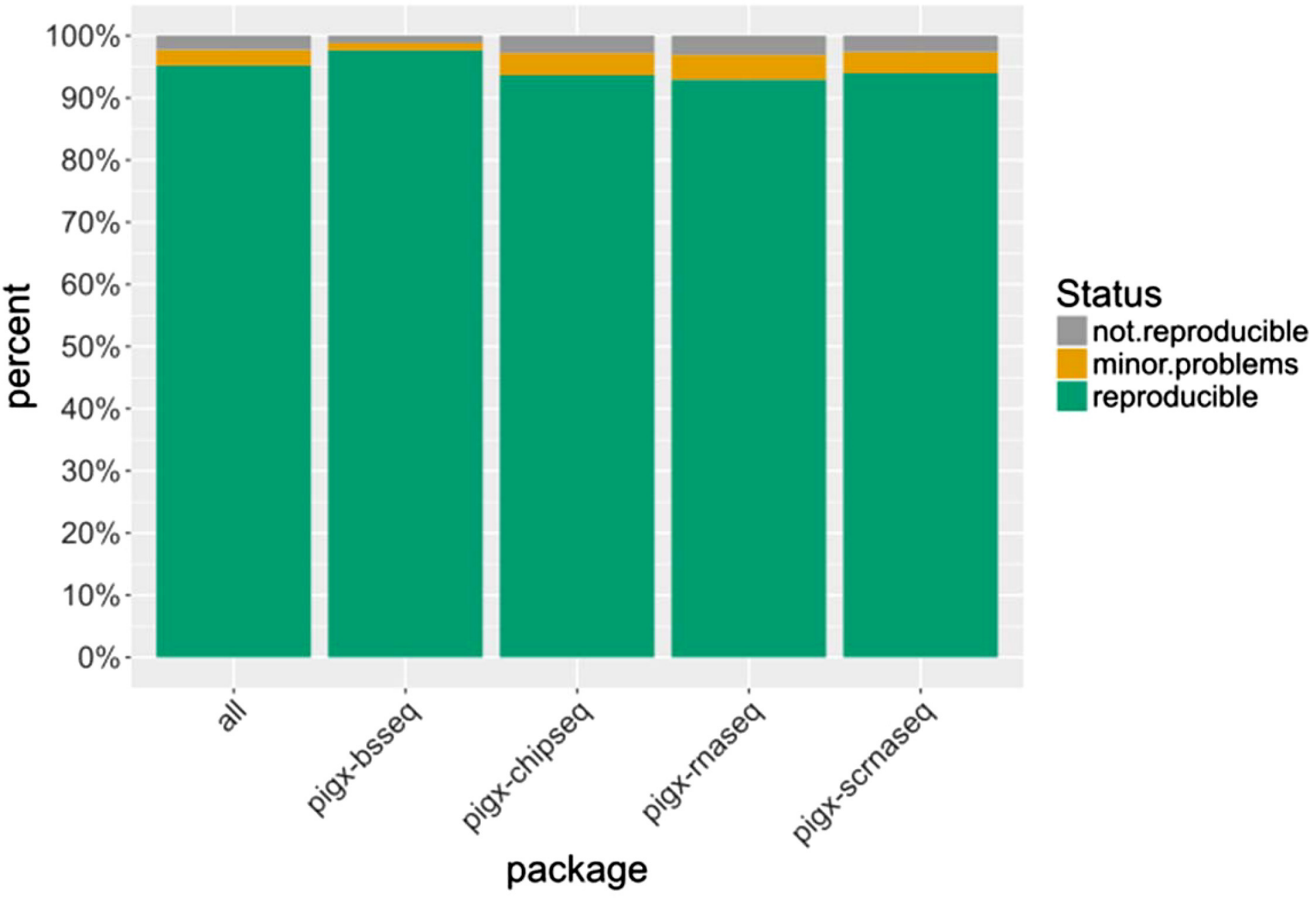
Percentage of directly dependent packages building in a bit-reproducible fashion across different systems for each of the pipelines.

Figure 9 shows the degree of bit reproducibility for the direct dependencies of each of the individual pipeline packages. Dependent packages whose files differed compared to builds on other systems fell either in the category of “minor problems” or “not reproducible,” dependent on the source and magnitude of nondeterminism. The exact dependency counts for each category and pipeline package are listed in Table 2. A comprehensive list of all dependent packages that were categorized as having “minor problems” is contained in Table 3. This table shows that the reproducibility problems of these packages are of negligible magnitude and could be corrected with minor patches to the package definitions in Guix.

**Table 3:** Packages with minor reproducibility problems and the magnitude of irreproducible files

Package	Magnitude	Notes
r-minimal	2 bytes	nondeterministic line break
python	∼6%	time stamp byte in header of byte code files
python-matplotlib	∼1.7%	single file difference
python-pycparser	∼3%	single file with time stamp
python-cffi	∼1.8%	recorded random test file names
python-numpy	<0.5%	six byte code files differ
python-simplejson	2 bytes	two files have single byte differences
gtk+	<1%	single file (icon cache)
glib	<0.1%	single file difference

### Alternative ways to install the pipelines

We generated application bundles containing all pipelines for use with Docker or Singularity. These container images were generated by exporting the “closure” (i.e., the package and all packages it references, recursively) of the pigx package (a package containing the individual pipeline packages pigx-bsseq, pigx-chipseq, pigx-rnaseq, and pigx-scrnaseq) from the declarative Guix package definition instead of iteratively modifying a base image containing a GNU+Linux operating system in a series of imperative steps. The container images are merely a translation of a functional description of the desired environment; as such, it is independent of global state, such as the contents of third-party package repositories or build time. The Docker image can be obtained at [43]; the Singularity image can be downloaded from [18]. We used Guix at commit 5149aeb7e62cf62398b55be38469cd28c25d8d7d (version v0.14.0–7054-g5149aeb7e) to generate these container images. This is the same version that we used to install the variant of PiGx with which the plots and reports in this publication were generated.

Since the pipelines use the well-known GNU build system as implemented by the Autotools suite, the pipelines can be configured and built in any environment providing the required dependencies. The portable configure script detects and records references to necessary software in the environment and reuses them at runtime using their absolute file names. Any package manager (such as Conda) can be used to fashion such a build-time environment. With regards to reproducibility, however, we recommend that a package manager be used that can provide separate, immutable, and uniquely prefixed environments to ensure that references to tools that are recorded at configuration time are identical to the variants that are used at runtime.

## Discussion

Computational workflows are becoming an indispensable part of the biological sciences as the field becomes more data intensive. The diversity and amount of data requires many tools for analysis. Consequently, published software or workflows often come with a complex set of dependencies. Even if sensible guidelines (e.g., [44]) such as sharing code online and providing documentation are employed, sometimes it is impossible to recreate the software used for analysis. Providing the code and documentation alone does not guarantee reproducibility or usability, nor do the Docker containers completely remedy this problem. In addition to reproducibility, there is also an increasing need for traceability and transparency, for the purposes of comprehensive data security in applications that manage the sensitive data collected in biomedical studies.

We propose GNU Guix and principled pipeline-as-software implementation as a solution to reproducibility problems in complex bioinformatics workflows. Here, we demonstrated the utility and reproducibility of the PiGx pipelines for genomics data analysis using GNU Guix.

Our decision to treat pipelines as first-class software packages and to adopt a conventional build system with Autotools made it possible to reduce the installation of complex software environments to a simple one-line command. By recording the exact locations of runtime dependencies of the pipeline packages during the configuration stage, we were able to eliminate ambiguity at runtime. When configuring the pipeline packages in an environment that ensures that different versions or variants of applications and libraries are stored in unique locations (such as an environment provided by GNU Guix), recording the exact location of dependencies at configuration time allows us to reproduce the detected environment at runtime.

We have shown that with a recursive definition of software dependencies using the framework provided by the functional package management paradigm as implemented in GNU Guix, it is possible to fully and exhaustively describe complex production-level bioinformatics software environments on GNU+Linux systems. The software environments were fully specified at the level of declarative, stateless package abstractions instead of using an imperative, stateful approach. We have also shown that the principled declarative approach to the management of software environments facilitates bit-reproducibility. The higher-level definitions of software environments can be translated in an automated fashion to lower-level application bundles such as Docker images. In contrast with container systems such as Docker or Singularity, Guix encloses the complete software environment and enables users to transparently rebuild it reproducibly from source without having to trust a binary application bundle. Due to referential transparency, binaries in Guix can only be the result of their corresponding sources.

Functional package management as implemented by GNU Guix significantly reduces the complexity of, and lowers the barrier to, managing bit-reproducible software environments. Users are freed from menial bookkeeping tasks such as keeping track of the origin of package binaries, the time of installation, the order of installation instructions, the state of the operating system at the time of installation, and any other runtime state. As far as users are concerned, it is enough to know the names of the packages that should be installed (in our case, simply “pigx”) and the current version of Guix; everything else such as source code provenance tracking, dependency management, package configuration, and compilation in isolated environments is handled by Guix. The guarantees provided by Guix enable users to analyze obstacles to experimental reproducibility beyond the software environment, such as sources of nondeterminism at runtime.

In our attempts to analyze the degree of repeatability of the HTML reports produced by PiGx, we identified a number of such sources of nondeterminism. The Salmon aligner, e.g., has a random component and does not provide a way for users to specify a seed for the pseudo-random number generators. This makes it impossible to exactly repeat an analysis and may require patching of the Salmon source code or virtualization of the random number generator facilities of the host system. Other tools are sensitive to the user's locale settings and may generate output in nondeterministic order. We were also surprised to find that an increasingly large number of tools rely on a connection to the Internet, either directly or indirectly, through dependent packages. This can be a great source of nondeterminism if the experimental setup does not take the volatile nature of networked resources into account.

Another important obstacle to reproducibility is the large kernel binary at runtime. Although the GNU C library provides a unified interface for all applications to use, the features that are actually implemented by the kernel at runtime may differ vastly. For example, the variant of Linux provided by Red Hat for their series 6 of operating systems reports its version as the obsolete and unsupported 2.6.32, but it contains many backported features from much newer kernel versions. Although this is usually not a problem, the kernel version and the implemented features should be taken into account. In order to make it possible to use the pipelines on Red Hat Enterprise Linux 6, we coordinated with other Guix developers to patch the GNU C library.

The use of a declarative mechanism to manage software environments is fundamental to comprehensive reproducibility. This encompasses repeatable builds, bit-reproducible binaries, software and data provenance, control over the configuration space, and deterministic runtime behavior. We have shown the feasibility of this approach in the domain of bioinformatics and propose that it serve as a template for reproducible computational workflows in other areas.

## Availability of source code and requirements

Project name: PiGx (pipelines in genomics)

Project home page: https://github.com/BIMSBbioinfo/pigx

Operating systems: any GNU/Linux system (kernel version > 3.10)

Programming languages: primarily GNU R and Python 3.

Other requirements: GNU Guix 0.15.0 or later for ready-made packages (if it is not possible to satisfy this requirement, Docker and Singularity images are provided)

License: GNU General Public License version 3, or (at your option) any later version.

RRID:SCR_016476

## Availability of supporting data

Snapshots of the code are available from the *GigaScience* GigaDB repository [45].

## Abbreviations

5hmC: 5-hydroxy-methyl-cytosine; 5mC: 5-methyl-cytosine; BS-seq: bisulfite-treated DNA sequencing; ChIP seq: chromatin immunoprecipitation sequencing; DNMT: DNA methyl-transferase; GO: Gene Ontology; GTF:Gene Transfer Format; HPC; high-performance computing; mESC: mouse embryonic stem cell; RNA-seq: RNA sequencing; scRNA-seq: single-cell RNA sequencing; TPM: transcripts per million reads; UMI: Unique Molecular Identifier

## Funding

B.U. acknowledges funding by the German Federal Ministry of Education and Research as part of the RNA Bioinformatics Center of the German Network for Bioinformatics Infrastructure (de.NBI; 031 A538C RBC). We also acknowledge support for K.W. from the Berlin Institute of Health. This project has received funding from the European Union's Horizon 2020 Research and Innovation Programme (under grant agreement 654248). Authors declare no competing interest.

## Supplementary Material

GIGA-D-18-00155_Original_Submission.pdf

GIGA-D-18-00155_Revision_1.pdf

GIGA-D-18-00155_Revision_2.pdf

Response_to_Reviewer_Comments_Original_Submission.pdf

Response_to_Reviewer_Comments_Revision_1.pdf

Reviewer_1_Report_(Original_Submission) -- Paolo Di Tommaso 05/25/2018 Reviewed

Reviewer_1_Report_Revision_1 -- Paolo Di Tommaso 6/8/2018 Reviewed

Reviewer_2_Report_(Original_Submission) -- Brad Chapman 5/25/2018 Reviewed

## Footnotes

1 The trailing x serves primarily as an aid to search specificity and denotes implementation using Gui**x**.
