# Supplementary material for: PiGx: reproducible genomics analysis pipelines with GNU Guix
Source: Gigascience. 2018 Oct 2;7(12):giy123. doi: 10.1093/gigascience/giy123 (PMC6275446; doi:10.1093/gigascience/giy123)
Supplement: Response_to_Reviewer_Comments_Original_Submission.pdf [file giy123_response_to_reviewer_comments_original_submission.pdf]

Author's Response To Reviewer Comments

Close

Reviewer #1: The authors introduce a method that enables reproducible genomic analyses based on GNU Guix, an open source package manager based on a functional/transactional paradigm.

The main strength of this method is the ability to capture the full graph of a data analysis dependencies both for the build and execution environments.

The manuscript is well written and easy to read, however there are some points that need to be clarified or reviewed:

* When discussing the usage of containers for data analysis reproducibility, the authors say: "Containers and binary disk images alone do not make traditional tooling any more suitable for the purpose of reproducible science". This statement does not provide an objective representation of the state of container technology. While containers are not a perfect solution, they have quickly become a reference solution to the problem of reproducibility. Several authors have shown how this technology can be used to successfully address the problem of reproducibility of complex data analysis workflows, see (1), (2), (3). Containers can provide the same level of bit-by-bit reproducibility as claimed by the method proposed by the authors (if not higher). The problem of transparency can be easily solved following best practices or using community collections such as BioContainers.

Response:
We thank the reviewer for their feedback and agree that greater clarity is needed regarding the benefits of our approach over containers. For many end-users, the appeal of 'reproducibility' is clear enough, and indeed, containers offer bit-for-bit identical execution binaries with 'reproducibility' in the same sense as ours. Traceability (e.g. from source to application bundle), is somewhat more subtle, however, and it is here that containers fail to offer any indication as to the origin of the bits that make up an application bundle.
Functional package management, on the other hand, offers referential transparency, whereby the full set of dependencies (and versions thereof) in a workspace are explicitly declared. Guix users need not rely on trust in developers' adherence to best practices to be confident of exactly which versions and sources are employed in a bundle, and to be certain that it is precisely these sources that have been used to generate their package. With Guix, there can be no undeclared dependencies, and malicious code cannot be hidden within an opaque binary bundle, but rather, must remain traceable to a specific source. Ref (A) shows the prevalence of potential security vulnerabilities in docker images, and the inheritance of such dependencies. This is of particular importance for data security in applications that have access to sensitive medical data, such as in bioinformatics. Indeed, as the authors of (1) state, Guix "represents the most rigorous approach towards dependency management".
Secondly, the authors of (2) suggest that the concept of continuous integration be applied to rerun experiments automatically whenever updates to the source code or data are applied. Since container description languages limit declaration of dependencies to other containers, however, undeclared dependencies would be ineffective as 'triggers', and it is not always clear when the re-execution of such experiments would be necessary.
Theoretically, container developers adhering to best-practices could maintain their containers' dependency lists; however, in practice, in large projects with many contributors, omission from human error becomes increasingly difficult to rule out. Guix ensures that dependencies are hard-

coded into a package’s construction, rather than relying on trust in 3rd parties.
We have added text to the manuscript to highlight this distinction between simple ‘reproducibility’, and referentially transparent reproducibility offered by Guix, along with the significance thereof.

* "Other package and environment managers .. fail to take the complete dependency graph into account, etc". This is a central point, the authors should provide a better description how the proposed method differs when compared to the other tools mentioned or provide a citation to sustain their claim.

Response:
As described in the above comments, Guix offers referential transparency, whereas containers depend on best-practices and judgement of the developer. Describing the detailed functional package management methodology behind Guix is beyond the scope of our work, but we agree that a citation is in order, and have added references to the works of Dolstra (2004) and Courtès (2015). The sentence in question has also been rephrased
* The authors put a lot of emphasis on the "bit-by-bit" reproducibility of the method proposed, however they conclude that it's not always possible due to non-deterministic build procedures, timestamp in the source files, tools relying on external components downloaded from the internet, etc. Maybe a better definition would be "near bit-by-bit reproducibility". At this regard it should be noted that containers allow real bit-by-bit reproducibility in the extend the resulting images are distributed in a binary format ie. do not require to re-compilation of the graph of the dependencies.

Response:
We agree greater clarity is needed on the differences in ‘reproducibility’ offered by containers and functional package management.
Containers and functional package management are complementary approaches. The former is a means of deploying existing binaries, the latter serves to generate those binaries. For example, containers built from Dockerfiles will differ substantially when built on different systems or at different times, as their files are commonly produced by non-deterministic processes, such as downloads from third-party package repositories or non-deterministic build processes.
Guix shows that we can build complex applications --- and thus even container images --- in a bit-reproducible fashion without having to sacrifice referential transparency or binary provenance (i.e. knowledge of the origin of said binary). From that point, containers produced by Guix can be copied, distributed and used without any re-compilation, and offers all the same functionality as container images generated via other means.
We have clarified the differences and the caveats that apply to reproducibility in the functional package management model in the manuscript.

* When discussing the reproducibility of the proposed method, it should be taken into account possible limiting factors. For example: the guix package is not usually available in common Linux distributions and its installation requires root permission. Also it's only available for the Linux operating system, therefore the applications depending on it cannot be deployed on different platforms. While this may not be a big problem for production scenarios, it can limit the application usage on computer platform commonly used for development and testing purpose. Finally, how accessible is a guix package definitions file, based on a functional notation, to an average user without knowledge of functional programming concepts and syntax?

Response:
It is true that, for the moment at least, Guix is only available on GNU+Linux systems. This has been now noted in the manuscript.
No knowledge of programming is required for the user to execute the pipelines described in the

manuscript; it is sufficient that users merely enter a few commands in a terminal for installation and execution (as described in the online documentation). Even so, they reap the benefit of reproducibility that is provided by functional package management.
The definition of custom Guix packages requires moderate programming expertise. Guix implements a domain specific language (DSL) that is embedded in the general purpose programming language Guile Scheme. Guix can also automatically convert package specifications written in JSON to its package DSL, and offers recursive importers, which generate package specifications automatically from a variety of third-party repositories such as CRAN, Bioconductor, CPAN, Pypi, Hackage, etc.
We would like to emphasize that the reproducibility of our method does not depend on users installing Guix or writing package definitions themselves. They can benefit from bit-reproducibility in other target formats, such as self-executing tarballs, SquashFS images for use with Singularity, or Docker images, although we suggest using Guix as a language with which to describe and understand software environments.
We have added a short note regarding the level of proficiency expected of the user, and the limitations of Guix-dependent workflows in the discussion.

* In the results is shown the usage of "pigx", however is not discussed what is this tool and why is needed.

Response:
Indeed, the original manuscript left this unclear, and we thank the reviewer for pointing out this oversight. The set of pipelines that we describe are collectively called “PiGx”, as an acronym for Pipelines in Genomics (where the ‘x’ is intended to aid the specificity of search results) We have clarified this in the manuscript.

* When discussing the reproducibility of the proposed method the authors provide metrics to assess the reproducibility of the graph of dependencies for the same pipeline deployed across three different systems. This is an interesting analysis, however it should also be provided a more detailed discussion and quantification of the *outputs* of the pipeline executions in different systems. It is mentioned that the repeatability was impacted by the non-determinism of some of the component used in the pipelines. Have they tried to compare the results of a pipeline not containing any source of non-determinism?

Response:
For a pipeline without any non-deterministic elements (in our case, the bs-seq pigx pipeline) the analysis is indeed repeatable, when a common time-stamp is supplied via the SOURCE_DATE_EPOCH environment variable, yielding bit-for-bit identical HTML reports on different machines. We have included a brief comment specifically addressing reproducibility of the reports.

* The authors should provide a detailed description how to replicate the execution of the data analysis pipelines described in the manuscript along with the used dataset.

Response:
Detailed description on the execution of the pipelines is provided in the online documentation, available here: http://bioinformatics.mdc-berlin.de/pigx_docs/.
Regarding the specific use-cases from the manuscript, we have now also supplied additional information (e.g. download sites, experimental accession IDs. etc.) in the supplementary materials.

1. Möller S., et al., Robust Cross-Platform Workflows: How Technical and Scientific Communities Collaborate to Develop, Test and Share Best Practices for Data Analysis,

https://link.springer.com/article/10.1007%2Fs41019-017-0050-4
2. Brett K Beaulieu-Jones & Casey S Greene, Reproducibility of computational workflows is automated using continuous analysis, 10.1038/nbt.3780
3. Di Tommaso P., Nextflow enables reproducible computational workflows, 10.1038/nbt.3820

Additional citation supplied to manuscript:
Rui Shu, Xiaohui Gu, and William Enck. 2017. A Study of Security Vulnerabilities on Docker Hub. In Proceedings of the Seventh ACM on Conference on Data and Application Security and Privacy (CODASPY '17). ACM, New York, NY, USA, 269-280. DOI: https://doi.org/10.1145/3029806.3029832

Reviewer #2: The authors describe a complete set of pipelines for RNA-seq, ChIP-seq, bisulfite sequencing and single cell RNA-seq. The focus of the pipelines is on ease of use and reproducibility, and they build on several existing tools: GNU guix for package installation, Snakemake for workflow execution and GNU autoconf to prepare and document the workflow system.

They then use these tools to walk through the implementations and show example analyses for the different pipelines. This is a great set of documentation and useful resource for the community.

Finally the authors describe an effort to characterize the reproducibility of the pipeline install to the level of hash-identical tools. This demonstrates that the hash-level issues are due to timestamps and other non-deterministic parts of binary builds affecting a small fraction of the tools.

This is a great initiative and demonstrates how to build reproducible pipelines making use of existing tooling. I have a couple of suggestions to help improve the paper:

- The major new initiative here is the use of Guix for binary compatibility. How do you feel this improves reproducibility over conda packages with pinned versions? You provide `requirements.txt` files in the GitHub repositories which look to represent this approach. How did you find they compare?

Response:
We thank the reviewer, Brad Chapman, for his time and feedback. Software version strings are prescriptive in the sense that they indicate only the intent of upstream developers to distinguish the source code of one version from any other version that they have authored previously. Version strings have limited descriptive power, as they fail to provide anything beyond a short name for a set of source files, and their descriptions are susceptible to human oversight. The configuration space (e.g. flags passed to the configure script or Makefiles), the state of the build-time environment (e.g. the compiler variant used to generate the binary), and dynamic linkage information (what exact library variants were linked with the binaries) are out of scope. This is appropriate, since version strings aren't intended to fully capture this state, but then version strings alone are insufficient to describe an application.
In the case of Conda, the current state of the Conda repositories remains undeclared, which precludes referential transparency (see discussion above) --although this could theoretically be approximated by maintaining a snapshot of the collection of Conda recipes and a well-defined, immutable build environment for all binaries. In practice, however, these recipes often refer to network resources; completely capturing the state of all such resources is infeasible.
With Guix the complete state for all packages is encoded in the state of the Guix source repository. There are no dependencies on the state of the system performing a package build. The build environment itself is reproducible without depending on opaque binary state.

- It would be worth mentioning alternative full stack alternatives to the workflow approach you're

taking. The most community driven one is Common Workflow Language plus a variety of runners. Right now this reads a bit as if you need Snakemake for the implementation, while in reality your approach with guix should work across multiple runners. What would it take in your opinion to utilize different workflow systems?

Response:
Indeed, Snakemake is not the only possible workflow framework that could be chosen for this particular set of pipelines; the choice of workflow framework is arbitrary. Snakemake was chosen primarily because it is already well-enough established, and compatible with a well-known programming language (i.e. Python). Thus, we felt the choice would be conducive to ease of use and adoption. We have added text to the manuscript to clarify the reasoning for this choice.

- Could you mention thoughts on maintainability of these pipelines over time? One of the hardest parts of building these types of integrated systems is continuing to develop and improve, which is where community engagement of existing solutions (bioconda, CWL) helps provide many hands to keep moving things forward. Do you feel that guix provides an advantage in terms of maintenance? How do you plan to support bugs and issues in previous versions as users go back to run older pipelines?

Response:
The collection of bioinformatics software in Guix has seen continued maintenance and development by people working in different institutions with a focus on bioinformatics. Although the number of regular contributors is probably smaller than the number of contributors to bioconda, it is growing and the community is actively inviting contributions and mentors newcomers, e.g. through Outreachy, GSoC, or internships at participating institutes. We are hopeful that the benefits provided by Guix will encourage more people in bioinformatics and other computationally intensive fields to adopt this approach.
The pipelines themselves are already being used for many collaborations within the Max Delbrueck Center. To broaden our user-base, our group also conducts regular training sessions for scientists who lack familiarity with computational bioinformatics at this institute and beyond. We encourage users of PiGx to contribute to the pipelines and share their experiences with us; to that end we have set up public source code repositories, a web site, and a public mailing list.

Close
