# Supplementary material for: PiGx: reproducible genomics analysis pipelines with GNU Guix
Source: Gigascience. 2018 Oct 2;7(12):giy123. doi: 10.1093/gigascience/giy123 (PMC6275446; doi:10.1093/gigascience/giy123)
Supplement: Response_to_Reviewer_Comments_Revision_1.pdf [file giy123_response_to_reviewer_comments_revision_1.pdf]

Author's Response To Reviewer Comments

Close

We carried out the formatting changes requested below.

>You probably need to decide whether you brand this GNU Guix or PiGx in the title too. Whatever you decide on you should probably mention both names in the abstract as this will be the only way people find it from searching in pubmed.

We are going with PiGx: Reproducible genomics analysis pipelines with GNU Guix. Abstract mentions both Guix and PiGx

>In addition, please register any new software application in the SciCrunch.org database to receive a RRID (Research Resource Identification Initiative ID) number, and include this in your manuscript. This will facilitate tracking, reproducibility and re-use of your tool.
>From an editorial perspective can you also add "Availability of source code" and "Availability of supporting data" sections at the end of your paper (just before the Acknowledgements). These should be formatted like this (please edit):
>Availability of supporting source code and requirements
>Project name: Pipelines in genomics
>Project home page: https://github.com/BIMSBbioinfo/pigx
>Operating system(s): e.g. Platform independent
>Programming language: e.g. Java
>Other requirements: e.g. Java 1.3.1 or higher, Tomcat 4.0 or higher
>License: e.g. GNU GPL, FreeBSD etc.
>RRID: if applicable, e.g. RRID: SCR_014986

This is done, we also obtained RRID.

>Availability of supporting data
>Snapshots of the code are available from the GigaScience GigaDB repository [Ref].
>Then in the references you need to cite the data in the paper (with the correct title) in the manner outlined:
>Wurmus R; Uyar B; Osberg B; Franke V; Gosdschan A; Wreczycka K; Ronen J; Akalin A (2018): Data supporting "PiGx: Pipelines in Genomics" GigaScience Database. http://dx.doi.org/10.5524/100501

This is done.

Close
