# Supplementary material for: PiGx: reproducible genomics analysis pipelines with GNU Guix
Source: Gigascience. 2018 Oct 2;7(12):giy123. doi: 10.1093/gigascience/giy123 (PMC6275446; doi:10.1093/gigascience/giy123)
Supplement: Reviewer_1_Report_(Original_Submission) -- Paolo Di Tommaso — 05/25/2018 Reviewed [file giy123_reviewer_1_report_(original_submission).pdf]

Reviewer Report

Title: **PiGx: Reproducible genomics analysis pipelines with GNU Guix**

Version: **Original Submission** **Date:** 5/25/2018

Reviewer name: **Paolo Di Tommaso**

Reviewer Comments to Author:

The authors introduce a method that enables reproducible genomic analyses based on GNU Guix, an open source package manager based on a functional/transactional paradigm.

The main strength of this method is the ability to capture the full graph of a data analysis dependencies both for the build and execution environments.

The manuscript is well written and easy to read, however there are some points that need to be clarified or reviewed:

* When discussing the usage of containers for data analysis reproducibility, the authors say: "Containers and binary disk images alone do not make traditional tooling any more suitable for the purpose of reproducible science". This statement does not provide an objective representation of the state of container technology. While containers are not a perfect solution, they have quickly become a reference solution to the problem of reproducibility. Several authors have shown how this technology can be used to successfully address the problem of reproducibility of complex data analysis workflows, see (1), (2), (3). Containers can provide the same level of bit-by-bit reproducibility as claimed by the method proposed by the authors (if not higher). The problem of transparency can be easily solved following best practices or using community collections such as BioContainers.

* "Other package and environment managers .. fail to take the complete dependency graph into account, etc". This is a central point, the authors should provide a better description how the proposed method differs when compared to the other tools mentioned or provide a citation to sustain their claim.

* The authors put a lot of emphasis on the "bit-by-bit" reproducibility of the method proposed, however they conclude that it's not always possible due to non-deterministic build procedures, timestamp in the source files, tools relying on external components downloaded from the internet, etc. Maybe a better definition would be "near bit-by-bit reproducibility". At this regard it should be noted that containers allow real bit-by-bit reproducibility in the extend the resulting images are distributed in a binary format ie. do not require to re-compilation of the graph of the dependencies.

* When discussing the reproducibility of the proposed method, it should be taken into account possible limiting factors. For example: the guix package is not usually available in common Linux distributions and its installation requires root permission. Also it's only available for the Linux operating system, therefore the applications depending on it cannot be deployed on different platforms. While this may not be a big problem for production scenarios, it can limit the application usage on computer platform commonly used for development and testing purpose. Finally, how accessible is a guix package definitions file, based on a functional notation, to an average user without knowledge of functional programming concepts and syntax?

* In the results is shown the usage of "pigx", however is not discussed what is this tool and why is needed.

* When discussing the reproducibility of the proposed method the authors provide metrics to assess the reproducibility of the graph of dependencies for the same pipeline deployed across three different systems. This is an interesting analysis, however it should also be provided a more detailed discussion and

quantification of the *outputs* of the pipeline executions in different systems. It is mentioned that the repeatability was impacted by the non-determinism of some of the component used in the pipelines. Have they tried to compare the results of a pipeline not containing any source of non-determinism?

* The authors should provide a detailed description how to replicate the execution of the data analysis pipelines described in the manuscript along with the used dataset.

Minor:
Page 28, line 15: "In our attemps" should be "In our attempt"

1. Möller S., et al., Robust Cross-Platform Workflows: How Technical and Scientific Communities Collaborate to Develop, Test and Share Best Practices for Data Analysis, https://link.springer.com/article/10.1007%2Fs41019-017-0050-4
2. Brett K Beaulieu-Jones & Casey S Greene, Reproducibility of computational workflows is automated using continuous analysis, 10.1038/nbt.3780
3. Di Tommaso P., Nextflow enables reproducible computational workflows, 10.1038/nbt.3820

Level of Interest

Please indicate how interesting you found the manuscript: Choose an item.

Quality of Written English

Please indicate the quality of language in the manuscript: Choose an item.

Declaration of Competing Interests

Please complete a declaration of competing interests, considering the following questions:

- Have you in the past five years received reimbursements, fees, funding, or salary from an organisation that may in any way gain or lose financially from the publication of this manuscript, either now or in the future?
- Do you hold any stocks or shares in an organisation that may in any way gain or lose financially from the publication of this manuscript, either now or in the future?
- Do you hold or are you currently applying for any patents relating to the content of the manuscript?
- Have you received reimbursements, fees, funding, or salary from an organization that holds or has applied for patents relating to the content of the manuscript?
- Do you have any other financial competing interests?
- Do you have any non-financial competing interests in relation to this paper?

If you can answer no to all of the above, write 'I declare that I have no competing interests' below. If your reply is yes to any, please give details below.

I declare that I have no competing interests

I agree to the open peer review policy of the journal. I understand that my name will be included on my report to the authors and, if the manuscript is accepted for publication, my named report including any attachments I upload will be posted on the website along with the authors' responses. I agree for my report to be made available under an Open Access Creative Commons CC-BY license (http://creativecommons.org/licenses/by/4.0/). I understand that any comments which I do not wish to be included in my named report can be included as confidential comments to the editors, which will not be published.

I agree to the open peer review policy of the journal

To further support our reviewers, we have joined with Publons, where you can gain additional credit to further highlight your hard work (see: https://publons.com/journal/530/gigascience). On publication of this paper, your review will be automatically added to Publons, you can then choose whether or not to claim your Publons credit. I understand this statement.

Yes
