# Supplementary material for: PiGx: reproducible genomics analysis pipelines with GNU Guix
Source: Gigascience. 2018 Oct 2;7(12):giy123. doi: 10.1093/gigascience/giy123 (PMC6275446; doi:10.1093/gigascience/giy123)
Supplement: Reviewer_2_Report_(Original_Submission) -- Brad Chapman — 5/25/2018 Reviewed [file giy123_reviewer_2_report_(original_submission).pdf]

Reviewer Report

Title **PiGx: Reproducible genomics analysis pipelines with GNU Guix**

Version: **Original Submission** **Date:** 5/25/2018

Reviewer name: **Brad Chapman**

Reviewer Comments to Author:

The authors describe a complete set of pipelines for RNA-seq, ChIP-seq, bisulfite sequencing and single cell RNA-seq. The focus of the pipelines is on ease of use and reproducibility, and they build on several existing tools: GNU guix for package installation, Snakemake for workflow execution and GNU autoconf to prepare and document the workflow system.

They then use these tools to walk through the implementations and show example analyses for the different pipelines. This is a great set of documentation and useful resource for the community.

Finally the authors describe an effort to characterize the reproducibility of the pipeline install to the level of hash-identical tools. This demonstrates that the hash-level issues are due to timestamps and other non-deterministic parts of binary builds affecting a small fraction of the tools.

This is a great initiative and demonstrates how to build reproducible pipelines making use of existing tooling. I have a couple of suggestions to help improve the paper:

- The major new initiative here is the use of Guix for binary compatibility. How do you feel this improves reproducibility over conda packages with pinned versions? You provide `requirements.txt` files in the GitHub repositories which look to represent this approach. How did you find they compare?

- It would be worth mentioning alternative full stack alternatives to the workflow approach you're taking. The most community driven one is Common Workflow Language plus a variety of runners. Right now this reads a bit as if you need Snakemake for the implementation, while in reality your approach with guix should work across multiple runners. What would it take in your opinion to utilize different workflow systems?

- Could you mention thoughts on maintainability of these pipelines over time? One of the hardest parts of building these types of integrated systems is continuing to develop and improve, which is where community engagement of existing solutions (bioconda, CWL) helps provide many hands to keep moving things forward. Do you feel that guix provides an advantage in terms of maintenance? How do you plan to support bugs and issues in previous versions as users go back to run older pipelines?

Thanks much for providing this great resource for users to learn about guix, reproducibility, and existing analysis pipelines. Hope these comments and thoughts are helpful in your work.

Brad Chapman <bchapman@hsph.harvard.edu>

Level of Interest

Please indicate how interesting you found the manuscript: Choose an item.

Quality of Written English

Please indicate the quality of language in the manuscript: Choose an item.

Declaration of Competing Interests

Please complete a declaration of competing interests, considering the following questions:

- Have you in the past five years received reimbursements, fees, funding, or salary from an organisation that may in any way gain or lose financially from the publication of this manuscript, either now or in the future?
- Do you hold any stocks or shares in an organisation that may in any way gain or lose financially from the publication of this manuscript, either now or in the future?
- Do you hold or are you currently applying for any patents relating to the content of the manuscript?
- Have you received reimbursements, fees, funding, or salary from an organization that holds or has applied for patents relating to the content of the manuscript?
- Do you have any other financial competing interests?
- Do you have any non-financial competing interests in relation to this paper?

If you can answer no to all of the above, write 'I declare that I have no competing interests' below. If your reply is yes to any, please give details below.

I declare that I have no competing interests

I agree to the open peer review policy of the journal. I understand that my name will be included on my report to the authors and, if the manuscript is accepted for publication, my named report including any attachments I upload will be posted on the website along with the authors' responses. I agree for my report to be made available under an Open Access Creative Commons CC-BY license (http://creativecommons.org/licenses/by/4.0/). I understand that any comments which I do not wish to be included in my named report can be included as confidential comments to the editors, which will not be published.

I agree to the open peer review policy of the journal

To further support our reviewers, we have joined with Publons, where you can gain additional credit to further highlight your hard work (see: https://publons.com/journal/530/gigascience). On publication of this paper, your review will be automatically added to Publons, you can then choose whether or not to claim your Publons credit. I understand this statement.

Yes
